# Constitutive overexpression of cellobiohydrolase 2 in *Trichoderma reesei* reveals its ability to initiate cellulose degradation

**DOI:** 10.1016/j.engmic.2022.100059

**Published:** 2022-11-15

**Authors:** Yubo Wang, Meibin Ren, Yifan Wang, Lu Wang, Hong Liu, Mei Shi, Yaohua Zhong

**Affiliations:** a State Key Laboratory of Microbial Technology, Microbial Technology Institute, Shandong University, Qingdao, 266237, China; b School of Medicine, Henan Polytechnic University, Jiaozuo, Henan 454003, China

**Keywords:** *Trichoderma reesei*, Cellobiohydrolase 2, Constitutive overexpression, Cellulose degradation, Cellulase induction

## Abstract

Cellulose degradation results from the synergistic effect of different enzymes, but which enzyme is involved in the initial stage of cellulose degradation is still not well understood. Cellobiohydrolase 2 (CBH2) attached to the conidial surface is possibly associated with the initial stage. However, its specific mechanism is still incompletely known. This study explored the potential role of CBH2 in initiating cellulose degradation using a constitutive overexpression strategy. First, the CBH2-overexpression *Trichoderma reesei* strains Qgc2–5 and Qrc2–40 were constructed using the constitutive promoters P*gpd1* and P*rpS30*, respectively. It was found that *cbh2* was expressed at a high level under the glucose conditions and was significantly higher than that of the parental strain QM9414 at the early stage of 29 h when cellulose was used as the carbon source. Particularly, the constitutive overexpression of *cbh2* caused the strong expression of major cellulase-encoding genes (*cbh1, eg1*, and *eg2*) and the rapid decomposition of cellulosic material. Meanwhile, the scanning electron microscope showed that the groove-like structure of the cellulose surface was eroded seriously owing to CBH2 overexpression, which caused the cellulose surface to be smooth. These results showed that the overexpression of CHB2 caused the major cellulase enzymes to be expressed and contributed to cellulose degradation, showing the potential role of CBH2 in the initial stage of the cellulose hydrolytic process.

## Highlights

• Overexpression of *cbh2* with different constitutive promoters in *Trichoderma reesei*.
• Cellobiohydrolase 2 promotes the early-stage expression of major cellulase genes.
• Cellobiohydrolase 2 contributes to the initiation of cellulose degradation.

## Graphical abstract

**Figure:**
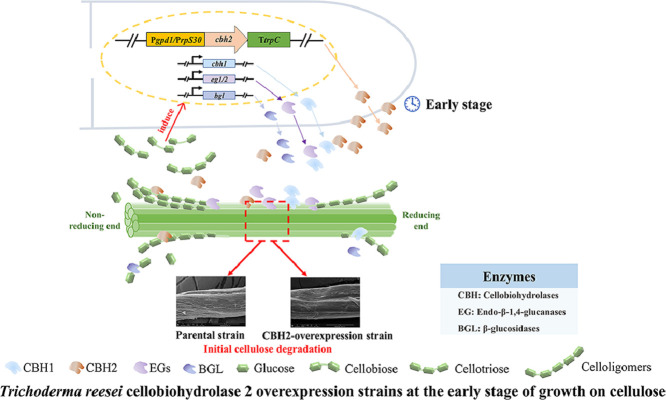

## 1 Introduction

Lignocellulose is the most abundant biomass resource on earth, comprising cellulose, hemicellulose, and lignin [1,2]. Among them, cellulose is the most abundant component of lignocellulose and comprises β−1,4-linked glucosyl units [1]. The hydrolysis of cellulose materials using cellulases to release fermentable sugars is important for converting cellulose to bioethanol. However, there is a problem in that cellulase cost is high, partly because of its low efficiency in degrading cellulosic materials.

The filamentous ascomycete *Trichoderma reesei* (*T. reesei*), as an industrial cellulase producer, has drawn much attention in years for its excellent ability to secrete a large number of cellulases [3,4], especially for converting lignocellulosic biomass to bioethanol [5] or other liquid fuels [6]. *T. reesei* could secrete many types of enzymes to degrade cellulose synergistically [7]. Cellobiohydrolases (CBHs, EC 3.2.1.91) hydrolyze the cellulose chain from the reducing end (by CBH1) or the non-reducing end (by CBH2) to manufacture cellobiose [8,9]; endoglucanases (EGs, EC 3.2.1.4) act on the non-crystalline region within cellulose and randomly cleave the β−1,4-glycosidic bond, exposing more CBH action sites [10]; and β-glucosidases (BGLs, EC 3.2.1.21) can hydrolyze cello-oligosaccharides to glucose in the final step [11]. CBHs are the most abundant enzymes in that CBH1 makes up 40%–60%, and CBH2 accounts for 20%–30% of the total number of secreted proteins in *T. reesei* [12,13]. Therefore, CBHs play a significant role in cellulose decomposition [14].

Although the expression level of CBH2 is lower than that of CBH1, it is catalytically more efficient [9,15]. It was reported that CBH2 could combine at least three contiguous β−1–4-linked glucosyl units to hydrolyze a glycosidic bond, thus having a higher substrate-specific activity toward crystalline cellulose than CBH1 [16]. Moreover, the CBH2 is efficiently heterologously expressed in *Pichia pastoris* [17], and the recombinant CBH2 enhances the enzymatic hydrolysis yield of cellulose by enhancing the exo-exo-synergism between CBH2 and CBH1 in *T. reesei* cellulase [18]. Recent studies have attempted to strengthen the total cellulase activity by increasing the CBH2 content in the secreted protein of *T. reesei*. The overexpression of the *cbh2* gene employing a *cbh1* strong promoter was conducted to enhance the production of cellobiohydrolases. The screened transformant C10 showed a 3.3-fold increase in filter paper activity and a 4.4-fold increase in cellobiohydrolase activity compared with the original strain *T. reesei* ZU-02 [19]. Qian et al. [20] constructed the CBH2 overexpression strain *T. reesei* QPC67 with the Double-joint PCR technique based on the *pyrG* marker, which caused the comparable secretion of CBH2 to CBH1; however, without significant changes in total cellulase activity compared with the original strain *T. reesei* QP4. Additionally, the influence of cellobiohydrolase deficiency was examined on the enzymatic hydrolysis of various cellulose substrates by constructing *cbh2*-deletion strains, and it was found that CBH2 might dominate the hydrolysis of crystalline cellulose [21]. However, the absence of *cbh2* only slightly affected the filter paper activity [21], [22], [23] and delayed cellulase formation on cellulose [24]. CBH1 particularly has drawn considerable attention [25], while the function of CBH2 is still quite unclear. It is known that CBH2 is important for the effective hydrolysis of cellulose and may be vital to the initiation of cellulase expression since it is the major component of cellulase located on the surface of spores [26,27]. The above research on CBH2 is summarized in Table 1.

**Table 1:** Research on the function and expression of *cbh2*^a^.

Host	Promoter	Type of promoter	Key strategies	Conclusion	Refs.
*T. reesei* ZU-02	*cbh1*	inducible	P*cbh1*-driven *cbh2* overexpression in *T. reesei* by *Agrobacterium*-mediated transformation.	The cellobiohydrolase production and the filter paper activity were greatly improved.	[19]
*Pichia pastoris*	*AOX1*	inducible	Expression of *T. reesei cbh2* in *P. pastoris* and addition of the recombinant CBH2 into *T. reesei* cellulases.	High enzymatic hydrolysis yields of SHPCS and SHPRS were obtained.	[18]
*P. pastoris*	*AOX1* & *GAP*	inducible & constitutive	Expression of *T. reesei cbh2* in *P. pastoris* with *AOX1* and *GAP* promoter, respectively.	The *AOX1* promoter was more suitable than the GAP for the CBH2 expression in *P. pastoris*.	[17]
*T. reesei* YH18	*cbh1*	inducible	P*cbh1*-driven *cbh2* overexpression in *T. reesei* and the On-site cellulase production using MML as an inducer.	Cellulase production was enhanced and the induction system was optimized.	[14]
*T. reesei* QP4	*cbh2*	inducible	Randomly inserted *cbh2* into the genome of *T. reesei* and investigated saccharification ability.	Overexpression of CBH2 does not markedly increase the saccharification ability.	[20]
*T. reesei* KU70	_	_	Disruption of *cbh2* in *T. reesei* and investigation of the enzymatic hydrolysis of different cellulose substrates.	The absence of CBH2 led to a significant reduction in the hydrolysis of microcrystalline cellulose.	[21]
*T. reesei* QM9414	*gpd1* & *rpS30*	constitutive	P*gpd1* or P*rpS30*-driven *cbh2* overexpression in *T. reesei* and analysis of structural changes on the cellulose surface.	CBH2 significantly contributes to the initial degradation of cellulose.	This study

a The abbreviations used in the table: MML, the soluble MGD (a mixture of glucose and β-disaccharide) and insoluble lignocellulosic residues; SHPCS, sodium hydroxide pretreated corn stover; SHPRS, sodium hydroxide pretreated rice straw; GAP, glyceraldehyde-3-phosphate dehydrogenase; AOX1, alcohol oxidase 1.

Cellulase expression relies on the induction by cellulose [28] or small molecular inducers, such as lactose [29,30] and sophorose [31]. Cellulose, as an insoluble inducible carbon source, cannot enter the cell through the cell membrane of *T. reesei* to induce cellulase secretion, but *T. reesei* can effectively degrade and use cellulose in nature [32]. It was reported that the CBH2 localized on the surface of conidia is essential for inducing cellulase by cellulose. For example, the content of CBH2 bound on the outer wall of conidia of the hyper-cellulase producer *T. reesei* Rut-C30 was higher than that of QM9414, which may contribute to the higher cellulase production by Rut-C30 [26]. Analysis of the relationship between *T. reesei* CBH2 and cellulase production was conducted mostly using the gene deletion strategy. Suominen et al. [22] reported that the deletion of the *cbh2* gene had minimal effects on the hydrolytic activities against hydroxyethylcellulose. Seiboth et al. [24] used a gene replacement strategy to generate *cbh2*-deletion strains. They discovered that the absence of CBH2 caused a considerable lag in the growth of cellulose and a delay in the formation of cellulases, demonstrating the role of CBH2 in the early growth phase of the fungus on cellulose. Furthermore, Seiboth et al. [33] revealed the importance of CBH2 for cellulase production in *T. reesei* at the transcriptional level. When cellulose was used as the sole carbon source, the deletion of *cbh2* caused the inability of *T. reesei* to express the other cellulase genes (*cbh1, eg1*, and *eg2*). Especially when *cbh1* and *cbh2* were knocked out, the fungus was utterly unable to grow under cellulose as the sole carbon source [21,33]. Nonetheless, there is no clear explanation yet for whether the *cbh2 gene* is involved in cellulase induction, mainly its function in degrading cellulose by *T. reesei*.

This study explores the relationship between CBH2 and cellulose degradation using the CBH2 overexpression strategy. Constitutive strong promoters were applied to drive *cbh2* expression in *T. reesei* to ensure high expression of *cbh2* during the initial stage of cellulose degradation. Furthermore, cellulase induction, cellulose consumption, and surface scanning electron microscopy (SEM) investigation was further combined to indicate the role of CBH2 during the cellulose degradation process.

## 2 Materials and methods

### 2.1 Strains and culture conditions

The *T. reesei* strain QM9414 (ATCC 26,921) was used for the *cbh2* gene overexpression experiments. The strains were maintained on potato dextrose agar (PDA) plates or slants supplemented. Sodium carboxymethyl cellulose (CMC-Na) plates were used for screening of overexpression CBH2 transformants, and the medium comprised CMC-Na 10.0-g∙L^−1^, (NH_4_)_2_SO_4_ 1.0-g∙L^−1^, Yeast extract 1.0-g∙L^−1^, MgSO_4_∙7H_2_O 0.5-g∙L^−1^, KH_2_PO_4_ 1.0-g∙L^−1^, and 2% (w/w) agar. The transparent zones of strains were observed after culturing at 30 °C for four days. To analyze the *cbh2* transcriptional level of the parental strain QM9414 on minimal medium (MM), conidia (10^8^∙-mL^−1^) were harvested and grown for 36, 48, 72, 96, and 120-h at 200-rpm and 30 °C in 500-mL Erlenmeyer flasks containing 100-mL MM [34]. Similarly, for CBH2-overexpression strains, conidia (10^8^∙-mL^−1^) were obtained and incubated for 48-h at 30 °C and 200-rpm. To analyze the transcriptional level of *cbh2* for the transformants at the early stage of cellulose degradation, pregrown mycelia of 0.75-g were transferred to 100-mL MM containing 1% Avicel (crystalline cellulose) as the sole carbon source and grown for an additional 4, 8, and 13-h. Then mycelia were obtained and used for RNA extraction. For cellulase activity assay, the strains were cultivated on seed medium [glucose 0.1-g∙L^−1^, CaCl_2_ 0.01-g∙L^−1^, MgSO_4_∙7H_2_O 0.006-g∙L^−1^, KH_2_PO_4_ 0.05-g∙L^−1^, (NH_4_)_2_SO_4_ 0.025-g∙L^−1^, and corn plasm 0.2-g∙L^−1^] for 36-h. Then, the mycelia were transferred into 100-mL cellulose-inducing medium [Avicel 0.2-g∙L^−1^, CaCl_2_ 0.01-g∙L^−1^, MgSO_4_∙7H_2_O 0.006-g∙L^−1^, KH_2_PO_4_ 0.05-g∙L^−1^, (NH_4_)_2_SO_4_ 0.025-g∙L^−1^, and corn plasm 0.2-g∙L^−1^] and cultured for five days.

### 2.2 Construction of the *T. reesei* cellobiohydrolase 2 constitutive overexpression strains

Double-joint PCR technique was used for constructing the transformants in this study, as described previously [35]. Expression cassettes were constructed for the overexpression of *cbh2* under the control of glyceraldehyde-3-phosphate dehydrogenase promoter (*Pgpd1*) in *T. reesei*. First, a 1.4-kb *gpd1* promoter and a 1.6-kb *cbh2* gene from *T. reesei* were amplified using the primer pairs *gpd1*-UF2/*gpd1*-UR3, *cbh2*-F1/*cbh2* + His8-R1, respectively. The 0.8-kb T*trpC* terminator was amplified by PCR using the primer pairs T*trpC* + His8-F1/T*trpC*-A with pAN7–1 as the template. Then these fragments were mixed and ligated by fusion PCR. Finally, a 2.8-kb hygromycin B resistance cassette *hph* was amplified using the primer pair *hph*-F2/*hph*-R2 with pAN7–1 as the template. Similarly, the *cbh2* overexpression cassette was constructed under the control of ribosomal protein S30 promoter (P*rpS30*) in *T. reesei*. For overexpression of *cbh2* under the control of P*rpS30*, firstly, a 1.3-kb *rpS30* promoter was amplified using primer pairs *rpS30*-UF1/*rpS30*-UR1. Then, the fragments of the *rpS30* promoter, *cbh2* coding region, and T*trpC* terminator were mixed and ligated by fusion PCR with nest pairs *rpS30*-UF2/T*trpC*-A. The primers used in this experiment are listed in Table 2.

**Table 2:** Primers used in this study.

Primers	Nucleotide sequence (5′→3′)	Purpose
*gpd1*-UF2	CAAGTTCTGGTCTCCGTTAAGG	*gpd1* promoter amplification
*gpd1*-UR3	GGTGAGAATGCCGACAATCATTTTGTATCTGAG GATTTGCGAGGCTG	
*cbh2*-F1	ATGATTGTCGGCATTCTCACC	*cbh2* amplification
*cbh2* + His8-R1	TTAGTGGTGGTGGTGGTGGTGGTGGTGCAGGA ACGATGGGTTTGCG	
*TtrpC +* His8*-*F1	CGCAAACCCATCGTTCCTGCACCACCACCACCACCACCACCACTAAACTTAACGTTACTGAAATCATCAAA	T*rpC* terminator amplification
T*trpC*-A	GAGTGGAGATGTGGAGTGGG	
*hph*-F2	ACAGAATAAGATAGGTGGAGAGC	hygromycin B gene amplification
*hph*-R2	CGAGTGGAGATGTGGAGTG	
*rpS30*-UF1	TTGGCGTAGCCTTTAGCAGTT	*rpS30* promoter amplification
*rpS30*-UR1	GGTGAGAATGCCGACAATCATGGTGATAGATTGATTTGGCGATG	
*gpd1-UF1*	CCTCTTCGGCGATACATACT	verification of *gpd1*
*cbh2-R1*	CTGCTAACTTCAGAGGCGTAA	verification of *cbh2*
*rpS30–338UF*	TACACGAACGGAAACTATGACG	verification of *rpS30*
*cbh1–575F*	GGTGGCGTGAGCAAGTATCC	qRT–PCR of *cbh1*
*cbh1–736R*	TGTCCTCCAATGCCCGTGTT	
*real-cbh2-S2*	CTGGTCCAACGCCTTCTTCA	qRT–PCR of *cbh2*
*real-cbh2-A2*	GACCCAGACAAACGAATCCAG	
*eg1*–491F	GGCTCGCTCTACCTGTCTCA	qRT–PCR of *eg1*
*eg1*–619R	GGGTGCCGTTCCTCCAT	
*eg2*–825F	ACGAGCCTTTGGTCGCAGTT	qRT–PCR of *eg2*
*eg2*–910R	GGCAGCCCAGGTGTTGATGT	
*cbh2-*probe*-*F	ACCCTCCTGGTTATTGGTATG	amplification of the probe of *cbh2*
*cbh2-* probe*-*R	GACCCAGACAAACGAATCCAG	

### 2.3 Total RNA extraction and quantitative real-time reverse transcription PCR

For RNA extraction, mycelia were harvested by filtration and homogenized in Mini-BeadBeater (Biospec, USA) with 0.5-mm zirconium/silica beads at 4 °C. Then total RNA was isolated with a TRIzol reagent Kit (Life Technologies, USA). The synthesis of cDNA from total RNA was conducted using PrimeScript® RT reagent Kit (Takara, Japan) as the manufacturer's protocol. Real-time PCRs were conducted in a Light Cycler 480 System (Roche Diagnostics, Germany). All PCRs were performed in triplicate in 20-µL reaction mixtures using the SYBR Premix Ex Taq™ (Tli RNaseH Plus) Kit (Takara, Japan). Light Cycler 480 software 1.5.0 was used to calculate the Ct value. Transcriptional levels of target genes were normalized against the level of the *actin* gene with ddCt technique. The sequences of the primers used for qRT–PCR are shown in Table 2.

### 2.4 Southern blotting

The probe of *cbh2* was a fragment amplified by PCR using the primer pair *cbh2*-probe-F and *cbh2*-probe-R to detect the *cbh2* gene. The genomic DNA digested through *Xho*Ⅰ was separated in 0.8% agarose gel and transferred to a Hybond-*N*^+^ nylon membrane (Amersham, USA). DNA labeling and detection were conducted using a DIG High Prime DNA Labeling and Detection Starter Kit I (Roche Diagnostics, Germany) according to the manufacturer's protocol.

### 2.5 Cellulase production and cellulose consumption

The total cellulase activity (filter paper activity, FPA) was measured using Whatman No.1 paper (Whatman, UK) as the substrate [36]. A specific measuring method was used as previously described [20]. One filter paper unit was defined as the amount of enzyme needed to decrease 1-μmol of sugar per minute. Because of the presence of insoluble cellulose and mycelia in culture, cellulose consumption could not be measured directly, but it could be measured by calculating the dry weight of mycelia indirectly. The dry weight of the mycelia was calculated using the equation between the intracellular protein content of the mycelia and the dry weight of the mycelia [37].

The specific operation method was to inoculate conidia (10^6^∙-mL^−1^) of QM9414 in MM, taking 5-mL and 10-mL fermentation broth into centrifuge tubes at 24, 36, and 48-h, respectively. The supernatant was removed after centrifugation at 10,000-rpm, and 4 °C for five minutes, and mycelia were rinsed using saline. After centrifugation twice, mycelia were obtained, and glass beads of 1/3 mycelial volume with 600-µL saline were added and shaken eight times in the Mini-Bead Beater for two minutes each time and ice bath for five minutes after each shaking immediately. Finally, the supernatants were obtained by centrifugation at 10,000-rpm and 4 °C for thirty minutes, and the intracellular protein concentration of each supernatant was determined using a Bio-rad DC Protein Assay Kit (Sangon Biotech, China). Simultaneously, 5-mL and 10-mL broth were obtained, respectively. Then, the mycelia were dried in an oven at 70 °C to constant weight, and the weight of the mycelia was weighed.

When the transformants were grown on the MM to the logarithmic stage, they were transferred to 150-mL MM with cellulose as the sole carbon source. At 29 and 48 h, the solid materials were obtained, and the total dry weight and intracellular protein concentration of the transformants were measured using the method described above.

### 2.6 Scanning electron microscope analysis of the surface structure of cellulose

At 0 and 29 h, 5-mL transformants fermentation broth of MM with cellulose as the sole carbon source was taken to obtain the mycelia mixture. Samples were fixed by adding 0.05-M sodium pyrophosphate solution at 4 °C overnight. After washing with 0.1-M phosphate buffer, the samples were placed in a boiling water bath for thirty minutes. Then, they were dehydrated for ten minutes, respectively, using a range of ethanol solutions from 10%, 30%, 50%, 70%, 90%, and 95%. Following, they were washed twice using 100% ethanol. In the end, the samples were dried overnight in the oven at 40 °C, and the surface morphology of the cellulose was observed under a scanning electron microscope (Thermo, USA) at a magnification of 2000 × and 8000 ×.

## 3 Results and discussion

### 3.1 Construction of the cellobiohydrolase 2 overexpression strains with constitutive promoters

In several eukaryotic microorganisms, the gene encoding glyceraldehyde-3-phosphate dehydrogenase (GPD) is constitutively and is high-level expressed. The promoter sequence of the *gpd*-encoding gene has been shown to be efficiently expressed in yeasts [38] and fungi [39,40] in homologous and exogenous genes. Therefore, the *gpd1* promoter of *T. reesei* was chosen to overexpress the *cbh2* gene. Additionally, the transcriptionally non-inducible RPS30 promoter was also used for this study [41,42]. Thus, two constitutive promoters, P*gpd1* and P*rpS30,* were used to drive the overexpression of CBH2 in this study to further examine the effect of CBH2 on cellulase induction and cellulose degradation.

**Fig. 1:**
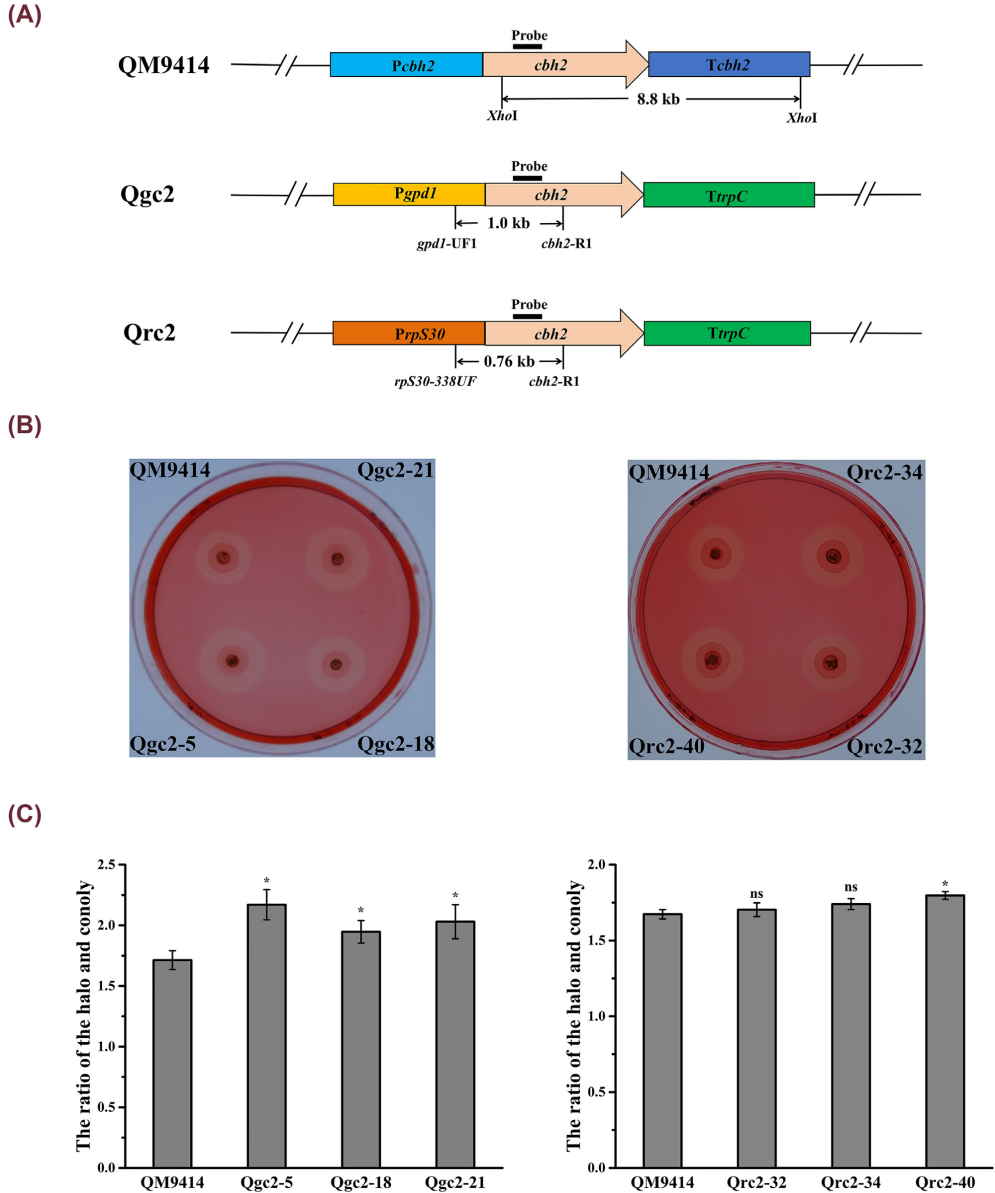
*Construction of the T. reesei CBH2-constitutive overexpression strains***.** (A) Schematic representation of the *cbh2* genomic loci from the transformants under the control of diverse promoters. Each gene cassette contains a promoter, a coding region, and a terminator. (B) The CBH2-constitutive overexpression strains were grown on a CMC-Na plate. (C) The ratio of the halo and colony. Data are shown as the mean of three independent experiments; error bars express the standard deviations. Comparisons between the parental strain and transformants were analyzed using Student's *t*-tests: *, *p* < 0.05; ns, not significant.

First, the *cbh2* expression cassettes, which contain the promoter *gpd1* or *rpS30*, the *cbh2* gene, and the *trpC* terminator, were constructed using Double-joint PCR. The two cassettes containing P*gpd1* and P*rpS30* were named Qgc and Qrc, respectively (Fig. 1A). Then, the individual *cbh2* expression cassette was co-transformed into *T. reesei* QM9414 with the *hph* expression cassette as a selection marker. The transformants were cultured in —CMC-Na plates for three days and screened. After staining using 1% Congo red and decolorization with 1-M NaCl, three Qgc transformants (Qgc2–5, Qgc2–18, and Qgc2–21) and three Qrc transformants (Qrc2–32, Qrc2–34, and Qrc2–40) were selected as they indicated significantly bigger transparent zones around the colonies than the parental strain QM9414 (Fig. 1B and C).

**Fig. 2:**
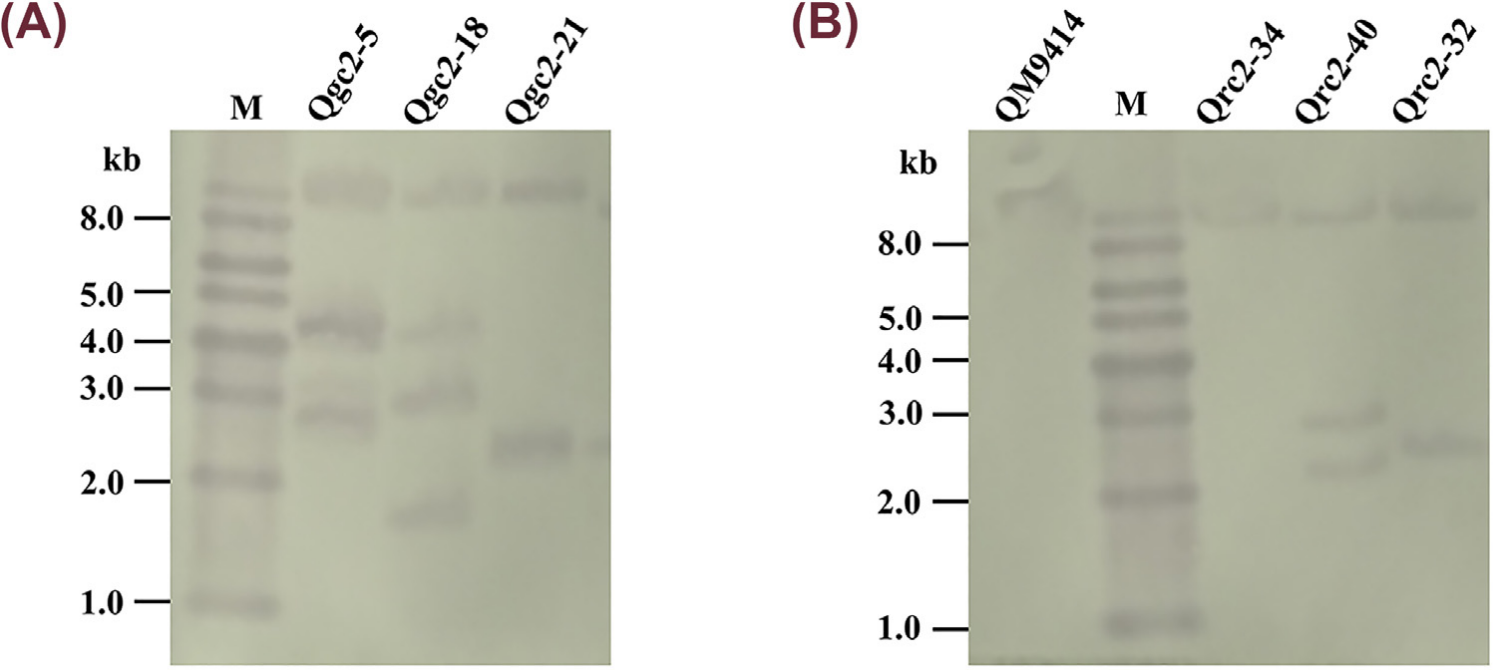
*Southern blotting of the CBH2-constitutive overexpression strains***.** (A) The overexpression strains with P*gpd1* as a promoter: Qgc2–5, Qgc2–18, and Qgc2–21. (B) The overexpression strains with P*rpS30* as a promoter: Qrc2–34, Qrc2–32, Qrc2–40, and the parental strain *T. reesei* QM9414.

To further confirm these candidate transformants, the *cbh2* expression cassettes were amplified from their genomes (data not shown). Then, Southern blot was employed to identify the copy number, and the *Xho*I-digested genomic DNA was hybridized through the *cbh2* probe (Fig. 2). An 8.8-kb fragment for the parental strain QM9414 was collected. In the Qgc transformants, two additional bands (2.8-kb and 4.2-kb) were observed for Qgc2–5, three other bands (1.7-kb, 2.8-kb, and 4.2-kb) were found for Qgc2–18, and an additional 2.3-kb band of Qgc2–21 was observed (Fig. 2A). As for the Qrc transformants, an additional 2.5-kb band was observed for Qrc2–32, and two additional bands (2.2-kb and 2.8-kb) were found for Qrc2–40 compared with the parental strain QM9414 (Fig. 2B). These results suggest that 1–4 copies of *cbh2* were successfully integrated into the genomes of transformants. It is worth noting that Qrc2–34 had only one 8.8-kb band, although the transparent zone radius of transformant Qrc2–34 was significantly larger than that of QM9414. This phenomenon may be because *Xho*I-digested the genomic DNA of Qrc2–34 to produce an additional 8.8-kb band that overlapped with the band produced by the parental strain QM9414. Finally, the CBH2-constitutive overexpression strains Qrc2–5 and Qgc2–40, which contain three copies of *cbh2* and have the largest radius of the transparent zone on their respective CMC—Na plates (Fig. 1B), were chosen to explore CBH2 function in cellulose degradation.

### 3.2 The high level of transcription of *cbh2* under glucose conditions

**Fig. 3:**
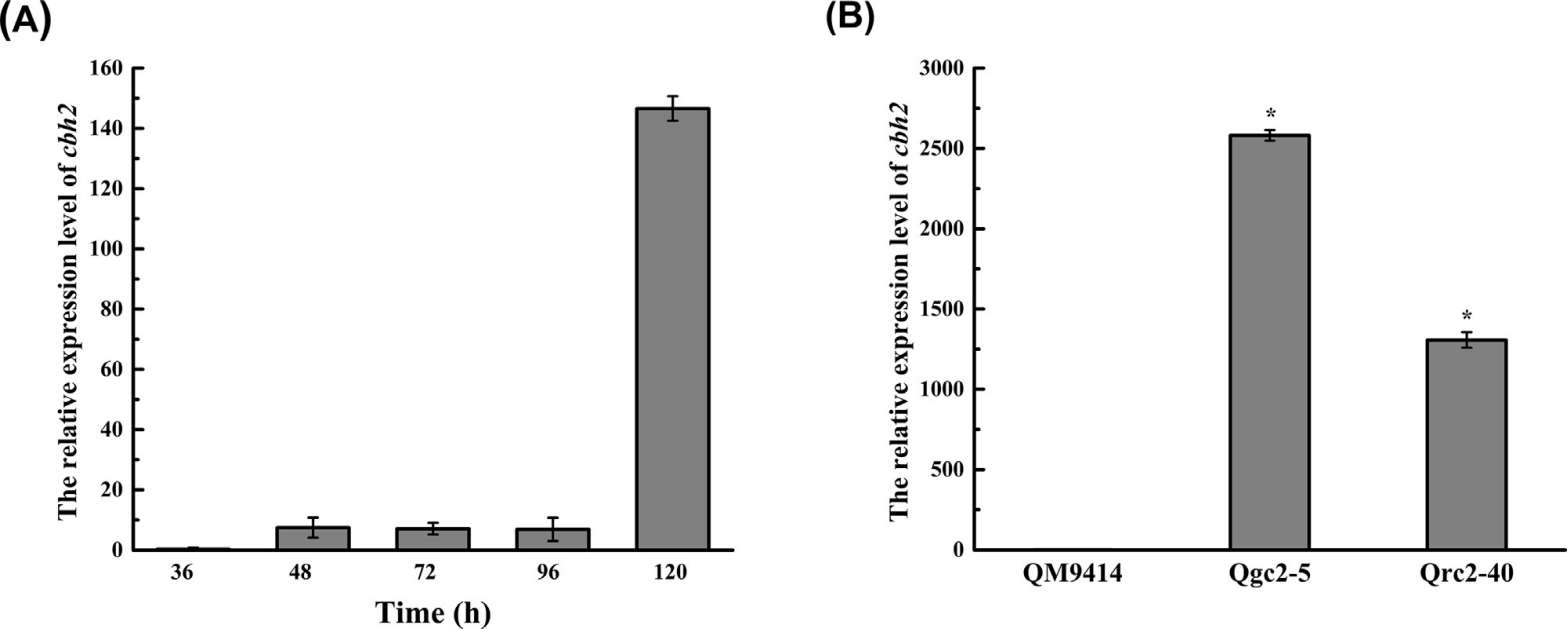
*Analysis of the transcript level of cbh2 on the MM plate containing glucose (2%) as the sole carbon source by qRT–PCR***.** (A) The transcriptional levels of *cbh2* for *T. reesei* QM9414 at different time points. (B) The transcript levels of the transformants Qgc2–5, Qrc2–40, and the parental strain QM9414 at 48-h. Comparisons between the parental strain and transformants were analyzed using Student's *t*-tests: *, *p* < 0.05.

*T. reesei* QM9414 exhibited the capability of cellulase expression only after glucose exhaustion when using glucose as the carbon source, which is because of the survival mechanism under starvation conditions [43]. CRE1 is associated with the regulation of *cbh2* [44,45], and it indirectly regulates the expression of *cbh2* through the regulation of the primary inducer Xyr1 [46]. When glucose is available, CRE1 preferentially uptakes readily metabolizable sugars (such as glucose and glycerol) and represses the expression of the genes encoding cellulase [47]. Thus, the cellulase-encoding genes of *T. reesei* were not expressed until glucose exhaustion. To detect the expression pattern of *cbh2*, qRT–PCR was first applied to assess the transcriptional level of *cbh2* in *T. reesei* QM9414 cultured in MM with glucose as the sole carbon source. It was found that the transcriptional level of *cbh2* was barely detectable in the early growth stage (before 48-h), and remained at a low level at 48–96-h, but enhanced significantly at 120-h (Fig. 3A). Considering that the CBH2-constitutive overexpression strains contain constitutive promoters, which can continuously express proteins without inducer restriction, these strains could have enough *cbh2* transcripts at 48 h to determine their transcriptional level. Thus, to investigate the *cbh2* expression efficiency using the promoters P*gpd1* and P*rpS30*, the optimal time to evaluate the expression level of *cbh2* for Qgc2–5 and Qrc2–40 in MM containing glucose as the sole carbon source was determined at 48-h. The results indicated that the transcriptional levels of *cbh2* in Qgc2–25 and Qrc2–40 were 3687-fold and 1868-fold, respectively, compared with that of the parental strain QM9414 (Fig. 3B), implying that the constitutive promoters P*gpd1* and P*rpS30* function in the CBH2-constitutive overexpression strains.

### 3.3 Constitutive overexpression of cellobiohydrolase 2 slightly elevates the cellulase production

**Fig. 4:**
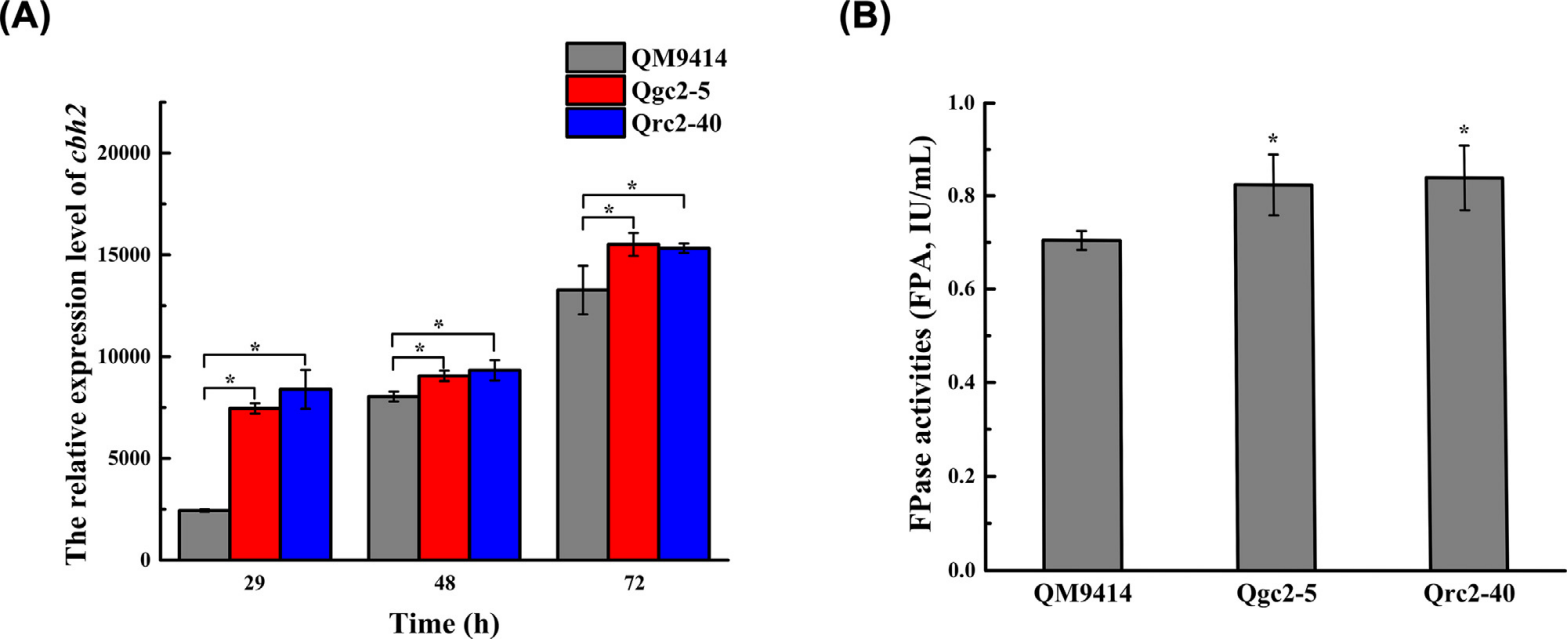
*Analysis of transcriptional levels of cbh2 and FPase activities under cellulose induction conditions***.** (A) The transcriptional levels of *cbh2* for the transformants Qgc2–5 and Qrc2–40 at 29, 48, and 72-h by qRT–PCR. (B) The FPase activities of the transformants Qgc2–5, Qrc2–40, and the parental strain *T. reesei* QM9414 at 72-h. Data are shown as the mean of three independent experiments; error bars indicate the standard deviations. Comparisons between the parental strain and transformants were analyzed using Student's *t*-tests: *, *p* < 0.05.

As CBH2 binds to the surface of the *T. reesei* conidia and is released during germination [48,49], it is hypothesized that it plays an important role in the initial cellulose degradation to facilitate the induction of cellulase components. Hence, the transcript abundance of the *cbh2* gene at 29, 48, and 72-h was assessed under cellulose induction conditions (Fig. 4A). As expected, the transcriptional levels of *cbh2* for Qgc2–5 and Qrc2–40 were 3.46-fold, and 3.92-fold higher than that of QM9414 at the early stage of 29 h. Whereas the transcriptional levels of *cbh2* in Qgc2–5 and Qrc2–40 were not obviously elevated compared with those of QM9414 at the late stages of 48 and 72 h. This is primarily due to the induction of high-level cellulase expression by cellulose in the early stage of cellulose degradation [3]. Then, the CBH2-constitutive overexpression strains Qgc2–5, Qrc2–40, and the parental strain QM9414 were inoculated in the cellulose-inducing medium at 30 °C and 200-rpm to test the total cellulase activity (FPA). The supernatants were obtained after 72-h, and the results are shown in Fig. 4B. The FPA of Qgc2–5 and Qrc2–40 were slightly improved compared with QM9414. Thus, the constitutive CBH2 overexpression has a positive effect on cellulase production in *T. reesei*.

### 3.4 Cellobiohydrolase 2 promotes the transcription of cellulase-encoding genes at the early stage of cellulose degradation

**Fig. 5:**
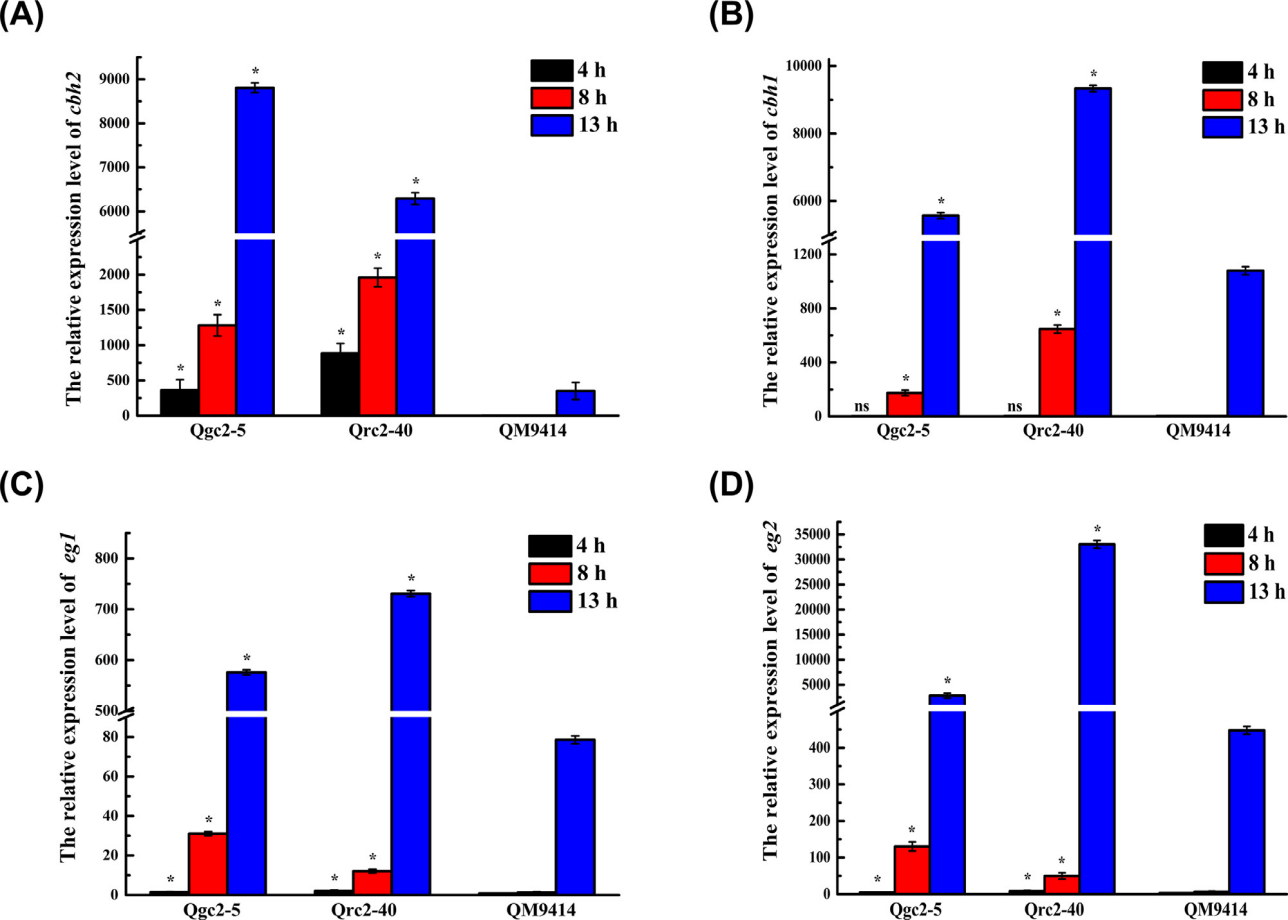
*Analysis of the relative expression levels of cbh2 (A), cbh1 (B), eg1 (C), and eg2 (D) when cellulose is used as the sole carbon source by qRT–PCR***.** Data are represented as the mean of three independent experiments; error bars express the standard deviations. Comparisons between the parental strain and transformants were analyzed using Student's *t*-tests: *, *p* < 0.05.

To further validate our hypothesis, the transformants were fermented in a cellulose-inducing medium, and the mycelia were harvested at a specified time interval. The transcriptional levels of the primary cellulase genes *cbh1, cbh2, eg1*, and *eg2* were determined. As indicated in Fig. 5, the expression of *cbh2* was found in Qgc2–5 and Qrc2–40 in four hours, while the expression of *cbh1, eg1,* and *eg2* was almost undetectable in QM9414. Interestingly, the transcriptional levels of *cbh2* in Qgc2–5 and Qrc2–40 in eight hours were approximately 64-fold and 238-fold higher than those of the parental strain QM9414, respectively; at 13-h, they were approximately 29-fold and 10-fold (Fig. 5A). Meanwhile, the expression levels of *cbh1, eg1,* and *eg2* in the transformants sharply increased from 4 to 13 h, which were significantly higher than that of QM9414 (Fig. 5B–D), showing that the constitutive overexpression of *cbh2* has remarkably promoted the expression of the major cellulase genes of *T. reesei* at the early stage of growth on cellulose.

### 3.5 Cellobiohydrolase 2 significantly contributes to the initial degradation of cellulose

To gain a better insight into the role played by CBH2 in the early stage of cellulose degradation, a further study was conducted to determine cellulose consumption. The mycelial dry weight was measured indirectly through the correspondence between intracellular protein content and mycelia produced. The linear relation equation between mycelial dry weight (*x*) and intracellular protein content (*y*) of QM9414 at various incubation times was obtained as *y* = 0.0814 *x* + 0.1396 (R^2^ = 0.993) using glucose as the carbon source (Supplementary data Fig. S1). Thus, the dry weight of mycelia was collected through this equation by measuring the intracellular protein content in the solid mixture.

**Fig. 6:**
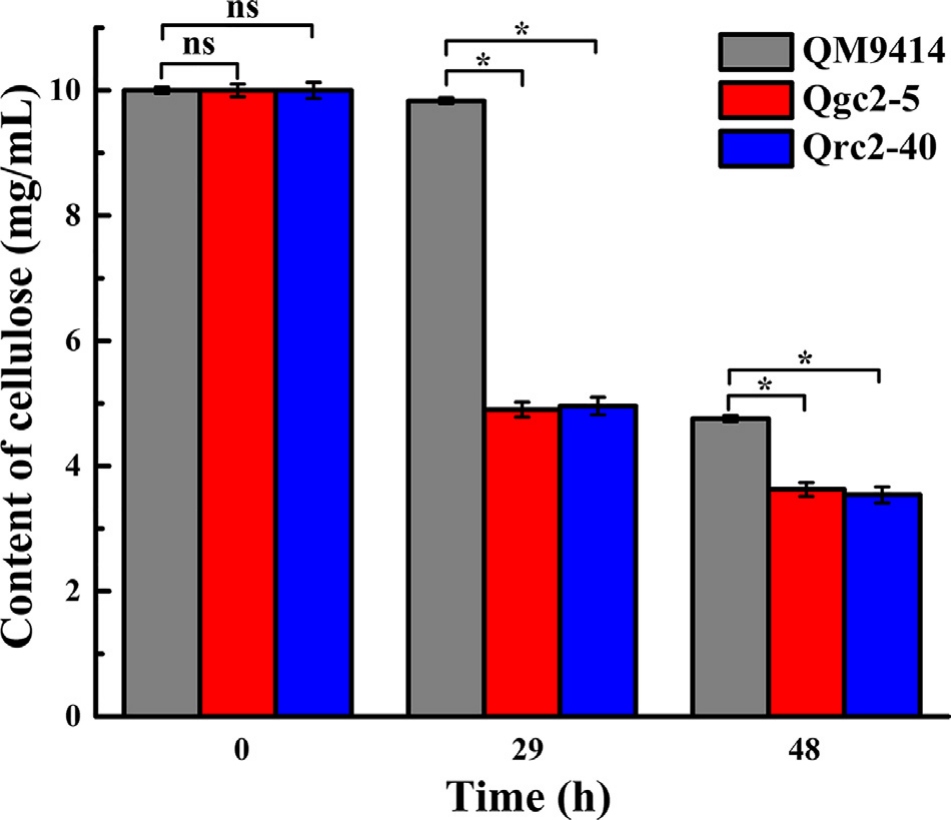
*The degradation of cellulose in the cellulose-inducing medium***.** The cellulose content in cellulose induction medium at 0, 29, and 48-h incubation for transformants Qgc2–5, Qrc2–40, and parental strain QM9414 are shown. Comparisons between the parental strain and transformants were analyzed using Student's *t*-tests: *, *p* < 0.05; ns, not significant.

The cellulose content remaining in the cellulose-inducing medium of culturing QM9414, Qgc2–5, and Qrc2–40 was calculated at 29 and 48-h by measuring the weight of the solid mixture and the intracellular protein content (Fig. 6). At 29 h, the cellulose content remaining in the culture medium of Qgc2–5 and Qrc2–40 was approximately half of the initial cellulose content, but the culture medium inoculated with QM9414 was barely degraded. At 48 h, only approximately 35% of the initial cellulose content remained in the culture medium inoculated with Qgc2–5 and Qrc2–40, and 48% of the initial cellulose content remained in the medium inoculated with QM9414. Thus, Qgc2–5 and Qrc2–40 indicated significantly faster cellulose degradation rates than QM9414 at the early stage of cellulose degradation, demonstrating that CBH2 played a significant role during the initial cellulose degradation, which was consistent with the conclusion obtained from the analysis of transcription. These results showed that the manipulation of the expression level of CBH2 could be used to increase cellulase production. Therefore, CBH2 can be overexpressed with constitutive promoters of various intensities to screen for the most effective promoter for the initial degradation of cellulose in future studies. Moreover, the CBH2 protein can be purified, and the purified CBH2 was supplemented into the cellulose-inducing medium, in which QM9414 was fermented to observe if it had the same effect on cellulose degradation as Qgc2–5 and Qrc2–40, thus demonstrating the exact CBH2 role during the process of cellulose degradation.

**Fig. 7:**
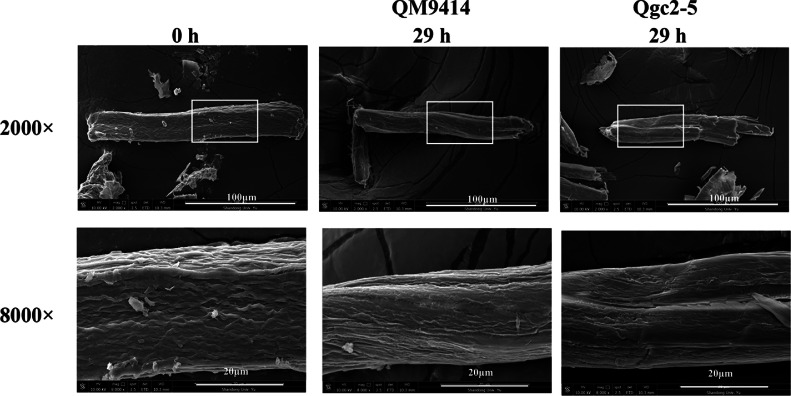
*Surface scanning electron microscopy (SEM) images of cellulose***.** SEM images of cellulose after 0 and 29-h hydrolysis using QM9414 and Qgc2–5 are shown.

To understand the molecular level changes that occurred in cellulose, Avicel inoculated with QM9414 and Qgc2–5 for 0 and 29 h was assessed using SEM at 2000 × and 8000 × magnification, respectively (Fig. 7). It could be clearly seen that the cellulose surface at 0-h has a regular groove-like structure as crystalline and non-crystalline regions alternate, showing that the cellulose surface is intact. At 29 h, the difference between the two samples of QM9414 and Qgc2–5 became clear. The degradation of cellulose by Qgc2–5 caused most of the cellulose surface to become smooth. However, the surface structure of cellulose through QM9414 was hydrolyzed to a lesser extent, where neatly arranged groove structures could still be noted in some areas. These results showed that CBH2 overexpression caused the cellulose surface structure to be seriously damaged at the early stage of cellulose degradation by *T. reesei*, providing evidence that CBH2 plays an important role during the cellulose degradation process.

## 4 Conclusions and future perspective

It has been recognized that CBH2 can initiate the growth of fungi on cellulose as it attacks cellulose chains from the non-reducing end to manufacture cellobiose, which could trigger the cascade reaction for cellulase synthesis [50,51]. Nevertheless, there is no direct evidence of CBH2funnction in the early stages of cellulose hydrolysis. In this study, the CBH2-constitutive overexpression strategy was adopted to explore CBH2’s role in cellulose degradation. The constitutive promoters P*gpd1* and P*rpS30* drove the high level of expression of *cbh2,* whether on glucose or cellulose as the sole carbon source. Overexpression of CBH2 triggered the induction of the major cellulase genes to be significantly elevated, which is probably attributable to the CBH2’s ability to begin the initial attack on cellulose to manufacture the cellulase inducers [33,52,53] and causes the cellulose surface to be heavily destroyed during the early stage of cellulose degradation.

Altogether, the results of this study will contribute to a better understanding of CBH2 function in the process of cellulosic material degradation by *T. reesei*, which has essential practical implications for the optimization of cellulase cocktails to further improve the saccharification efficiency. Furthermore, these data will facilitate the construction of industrial strains for efficient cellulose degradation, which may trigger the economical competition for cellulosic biorefinery.

## Declaration of Competing Interest

The authors declare that they have no known competing financial interests or personal relationships that could have appeared to influence the work reported in this paper.

## Appendix Supplementary materials

Image, application 1
