# Supplementary material for: Constitutive overexpression of cellobiohydrolase 2 in Trichoderma reesei reveals its ability to initiate cellulose degradation
Source: Eng Microbiol. 2022 Nov 15;3(1):100059. doi: 10.1016/j.engmic.2022.100059 (PMC11611025; doi:10.1016/j.engmic.2022.100059)
Supplement: Supplementary file 1 [file mmc1.docx]

## Supplement materials

**Constitutive overexpression of cellobiohydrolase 2 in** ***Trichoderma reesei* reveals its ability to initiate cellulose degradation**

Yubo Wang^a,1^, Meibin Ren^b,1^, Yifan Wang^a^, Lu Wang^a^, Hong Liu^a^, Mei Shi^a,*^, clubstone@sdu.edu.cn, Yaohua Zhong^a,*^, [zhongyaohua@sdu.edu.cn](mailto:zhongyaohua@sdu.edu.cn)

^a^ State Key Laboratory of Microbial Technology, Microbial Technology Institute, Shandong University, Qingdao, Shandong 266237, China

^b^ School of Medicine, Henan Polytechnic University, Jiaozuo, Henan 454003, China

Running head: Cellobiohydrolase 2 contributes to initiating cellulose degradation

*Correspondence authors.

^1^These authors contributed equally to this work.


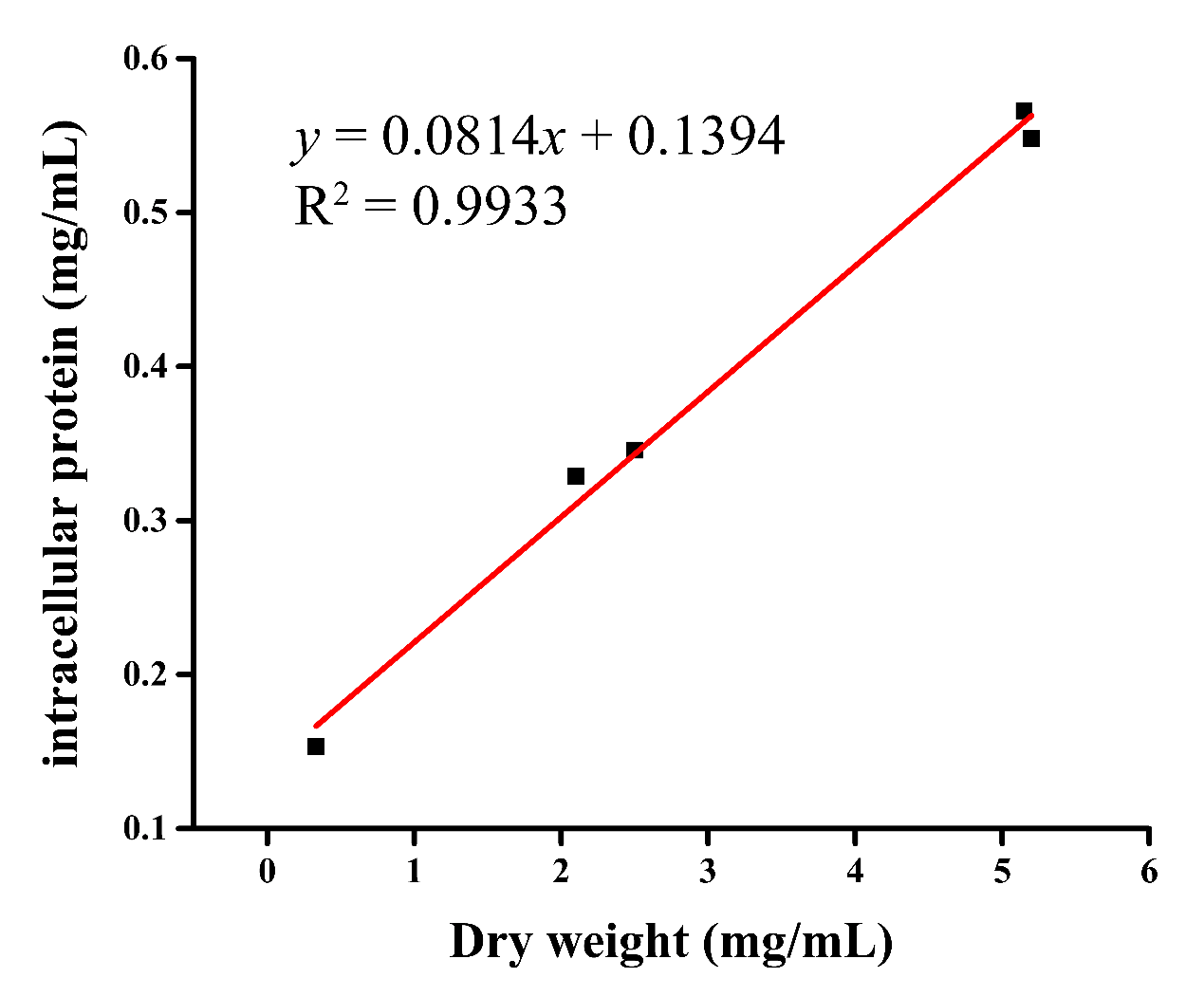


**Fig. S1.** The linear relation equation between intracellular mycelial dry weight and protein content of QM9414 at different incubation time.
